# Author Correction: TFPa/HADHA is required for fatty acid beta-oxidation and cardiolipin re-modeling in human cardiomyocytes

**DOI:** 10.1038/s41467-020-16186-9

**Published:** 2020-05-08

**Authors:** Jason W. Miklas, Elisa Clark, Shiri Levy, Damien Detraux, Andrea Leonard, Kevin Beussman, Megan R. Showalter, Alec T. Smith, Peter Hofsteen, Xiulan Yang, Jesse Macadangdang, Tuula Manninen, Daniel Raftery, Anup Madan, Anu Suomalainen, Deok-Ho Kim, Charles E. Murry, Oliver Fiehn, Nathan J. Sniadecki, Yuliang Wang, Hannele Ruohola-Baker

**Affiliations:** 1 Institute for Stem Cell and Regenerative Medicine, University of Washington, School of Medicine, Seattle, WA 98109 USA; 2 Department of Bioengineering, University of Washington, Seattle, WA 98195 USA; 3 Department of Biochemistry, University of Washington, School of Medicine, Seattle, WA 98195 USA; 4 Department of Mechanical Engineering, University of Washington, Seattle, WA 98195 USA; 5 Center for Cardiovascular Biology, University of Washington, Seattle, WA 98109 USA; 6 NIH West Coast Metabolomics Center, University of California Davis, Davis, CA 95616 USA; 7 Department of Pathology, University of Washington, Seattle, WA 98109 USA; 8 Helsinki University Hospital, 00290 Helsinki, Finland; 9 Research Programs Unit, Stem Cells and Metabolism, University of Helsinki, 00290 Helsinki, Finland; 10 Department of Anesthesiology and Pain Medicine, Mitochondria and Metabolism Center, University of Washington, Seattle, WA 98109 USA; 11 Covance Genomics Laboratory, Redmond, WA 98052 USA; 12 Neuroscience Center, University of Helsinki, 00290 Helsinki, Finland; 13 Department of Medicine/Cardiology, University of Washington, Seattle, WA 98109 USA; 14 Biochemistry Department, Faculty of Science, King Abdulaziz University, Jeddah, Saudi Arabia; 15 Paul G. Allen School of Computer Science & Engineering, University of Washington, Seattle, WA 98195 USA

**Keywords:** Mechanisms of disease, Cardiovascular diseases

Correction to: *Nature Communications* 10.1038/s41467-019-12482-1, published online 11 October 2019

The original version of the Supplementary Information associated with this Article did not include the Supplemental Tables. The HTML has been updated to include a corrected version of the Supplementary Information. In addition, Supplemental Table S11 was not mentioned in the following sentence, where it should have been referred to together with Supplemental Table S10: “Cluster 1 showed an intermediate state of CM maturity, characterized by elevated OXPHOS and Myc target genes (Supplemental Table S10)”. This reference has now been added both in the PDF and HTML versions of the Article.

