# Supplementary material for: TFPa/HADHA is required for fatty acid beta-oxidation and cardiolipin re-modeling in human cardiomyocytes
Source: Nat Commun. 2019 Oct 11;10:4671. doi: 10.1038/s41467-019-12482-1 (PMC6789043; doi:10.1038/s41467-019-12482-1)
Supplement: Supplementary file 1 — Supplementary Information [file 41467_2019_12482_MOESM1_ESM.pdf]

TFPa/HADHA is required for fatty acid beta-oxidation and cardiolipin re-modeling in human cardiomyocytes

Miklas et al.

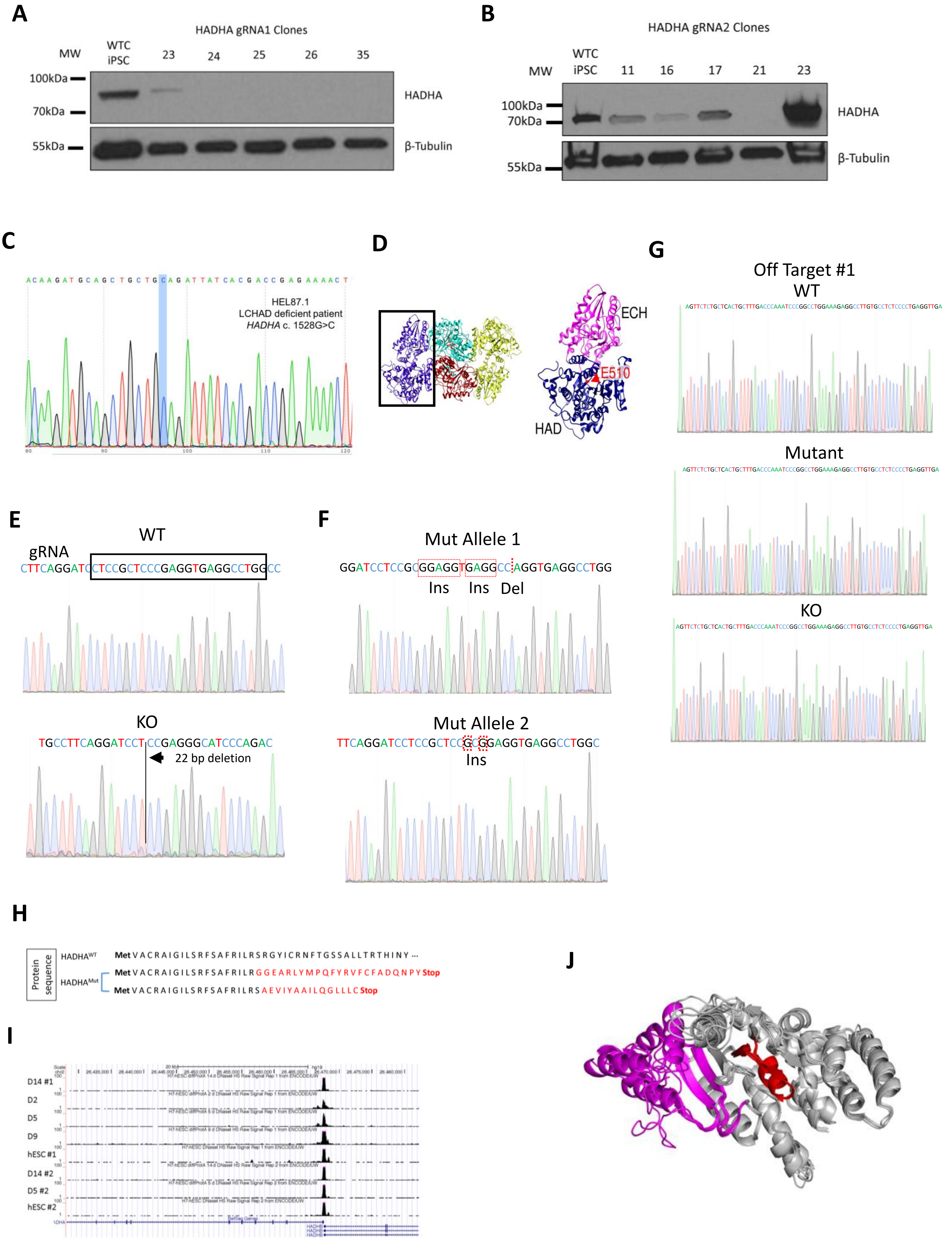
A
HADHA gRNA1 Clones
MW
WTC iPSC
23
24
25
26
35
100kDa
70kDa
55kDa
HADHA
β-Tubulin
B
HADHA gRNA2 Clones
MW
WTC iPSC
11
16
17
21
23
100kDa
70kDa
55kDa
HADHA
β-Tubulin
C
HEL87.1
LCHAD deficient patient
HADHA c. 1528G>C
D
ECH
E510
HAD
G
Off Target #1
WT
Mutant
KO
E
WT
gRNA
CTTCAGGATCCTCCGCTCCCGAGGTGAGGCCTGGCC
KO
TGCCTTCAGGATCCTCCGAGGGCATCCCAGAC
22 bp deletion
F
Mut Allele 1
GGATCCTCCGCGGAGGTGAGGCCAGGTGAGGCCTGG
Ins
Ins
Del
Mut Allele 2
TTCAGGATCCTCCGCTCCGCGGAGGTGAGGCCTGGC
Ins
H
Protein sequence
HADHA^WT
Met V A C R A I G I L S R F S A F R I L R S R G Y I C R N F T G S S A L L T R T H I N Y ...
HADHA^Mut
Met V A C R A I G I L S R F S A F R I L R G G E A R L Y M P Q F Y R V F C F A D Q N P Y Stop
Met V A C R A I G I L S R F S A F R I L R S A E V I Y A A I L Q G L L L C Stop
I
D14 #1
D2
D5
D9
hESC #1
D14 #2
D5 #2
hESC #2
J

Supplemental Figure 1: HADHA iPSC clone analysis. A) Western of five clones generated from gRNA1 induction of HADHA mutations. B) Western of five clones generated from gRNA2 induction of HADHA mutations. HADHA iPSC mutation characterization. C) Chromographs of c.1528G>C *HADHA* hiPSCs (HEL87.1) showing the point mutation. D) Protein model of HADHA, highlighting the domain and position of the mutated amino acid (E510) in the enzyme region HAD of HADHA. E) Chromographs of WTC iPSC WT and HADHA gRNA1 clone #35 (KO). F) Chromograph of clone #23 (mutant, each allele shown). WT iPSC shows the gRNA region in the red box. KO shows the position of the 22bp deletion. G) Chromographs of WTC iPSC WT, HADHA mutant and HADHA KO off-target #1 PCR amplification. H) Predicted protein sequence of HADHA Mut (clone #23). I) DNA-Seq. data showing the location of H3K4me3 marks indicating transcription start site. HADHA is expressed in the stem cell state and throughout stem cell derived cardiomyocyte differentiation at D2, 5, 9 and 14 with only one region in exon 1 for the TSS. J) HADHA protein model of exons 1-11 (half the protein) showing exons 1-3 in purple, exons 4-11 in grey and the enoyl-CoA hydratase catalytic pocket in red. Source data are provided as a Source Data file.

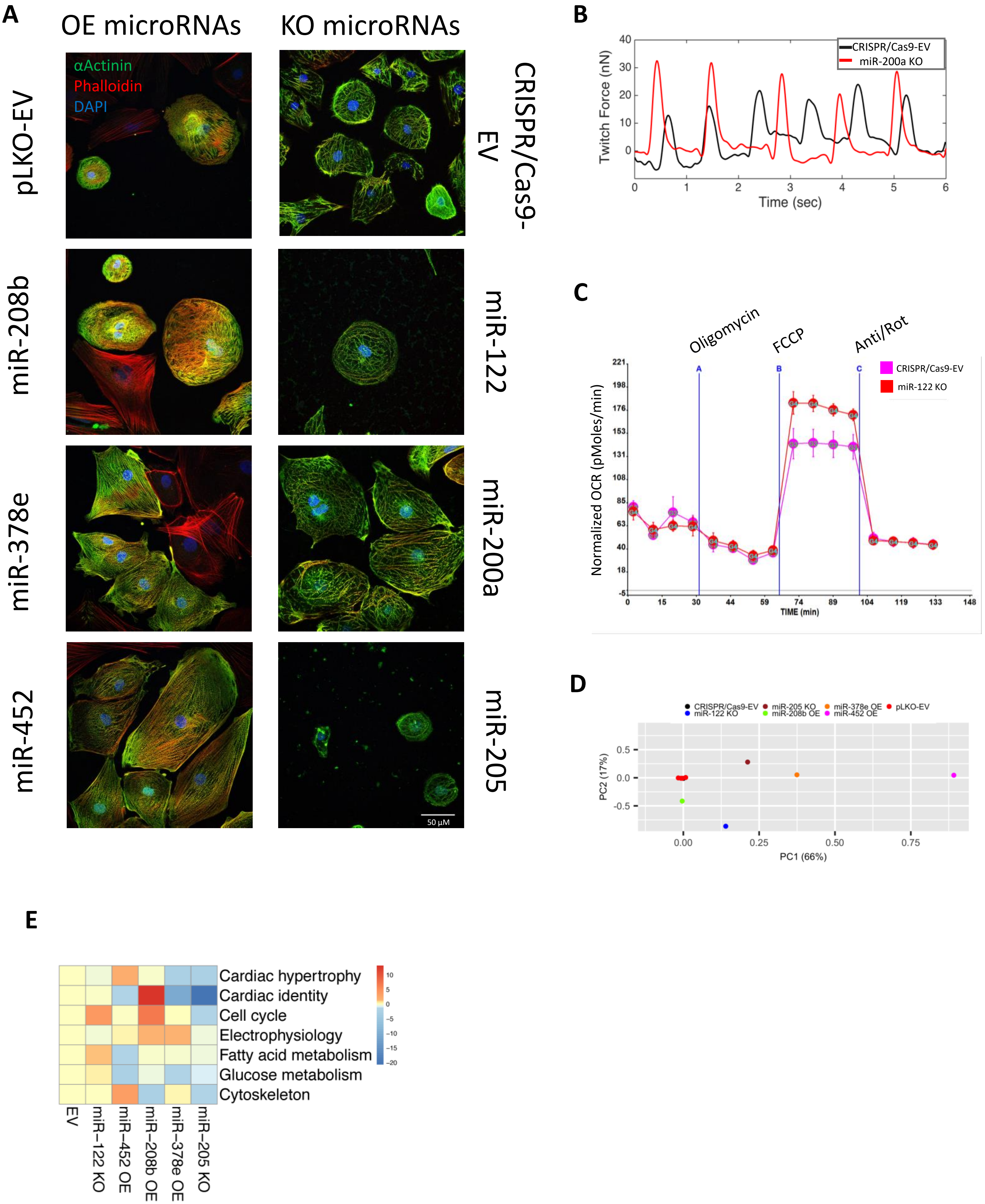
A
OE microRNAs
KO microRNAs
αActinin
Phalloidin
DAPI
pLKO-EV
CRISPR/Cas9-EV
miR-208b
miR-122
miR-378e
miR-200a
miR-452
miR-205
50 μM
B
CRISPR/Cas9-EV
miR-200a KO
Twitch Force (nN)
Time (sec)
C
Oligomycin
FCCP
Anti/Rot
CRISPR/Cas9-EV
miR-122 KO
Normalized OCR (pMoles/min)
TIME (min)
D
CRISPR/Cas9-EV
miR-122 KO
miR-205 KO
miR-208b OE
miR-378e OE
miR-452 OE
pLKO-EV
PC2 (17%)
PC1 (66%)
E
Cardiac hypertrophy
Cardiac identity
Cell cycle
Electrophysiology
Fatty acid metabolism
Glucose metabolism
Cytoskeleton
EV
miR−122 KO
miR−452 OE
miR−208b OE
miR−378e OE
miR−205 KO

Supplemental Figure 2: Identification of microRNAs that govern the maturation of hPSC-CMs. A) Representative confocal microscopy images for the six screened miRs and their respective controls. Green denotes the cardiac structural protein αActinin, red denotes phalloidin which stains F-actin and blue denotes DAPI which stains cell nuclei. B) Representative force trace for a control hPSC-CM and a miR-200a KO treated hPSC-CM. C) KO of miR-121 led to a significant increase in maximum oxygen consumption rate. Representative trace of the mitostress assay is shown. Error bars show standard deviation. D) Principal component analysis (PCA) of RNA-Sequencing data for the OE or KO of a single microRNA in Day-30 hPSC-CMs. E) Heatmap showing seven pathways that are associated with hPSC-CM maturation and how each individual microRNA's perturbation of hPSC-CM transcriptome influences the expression of each pathway. Red indicates genes up-regulated by the treatment show more significant enrichment in the pathway than genes down-regulated by the treatment, blue means the opposite.

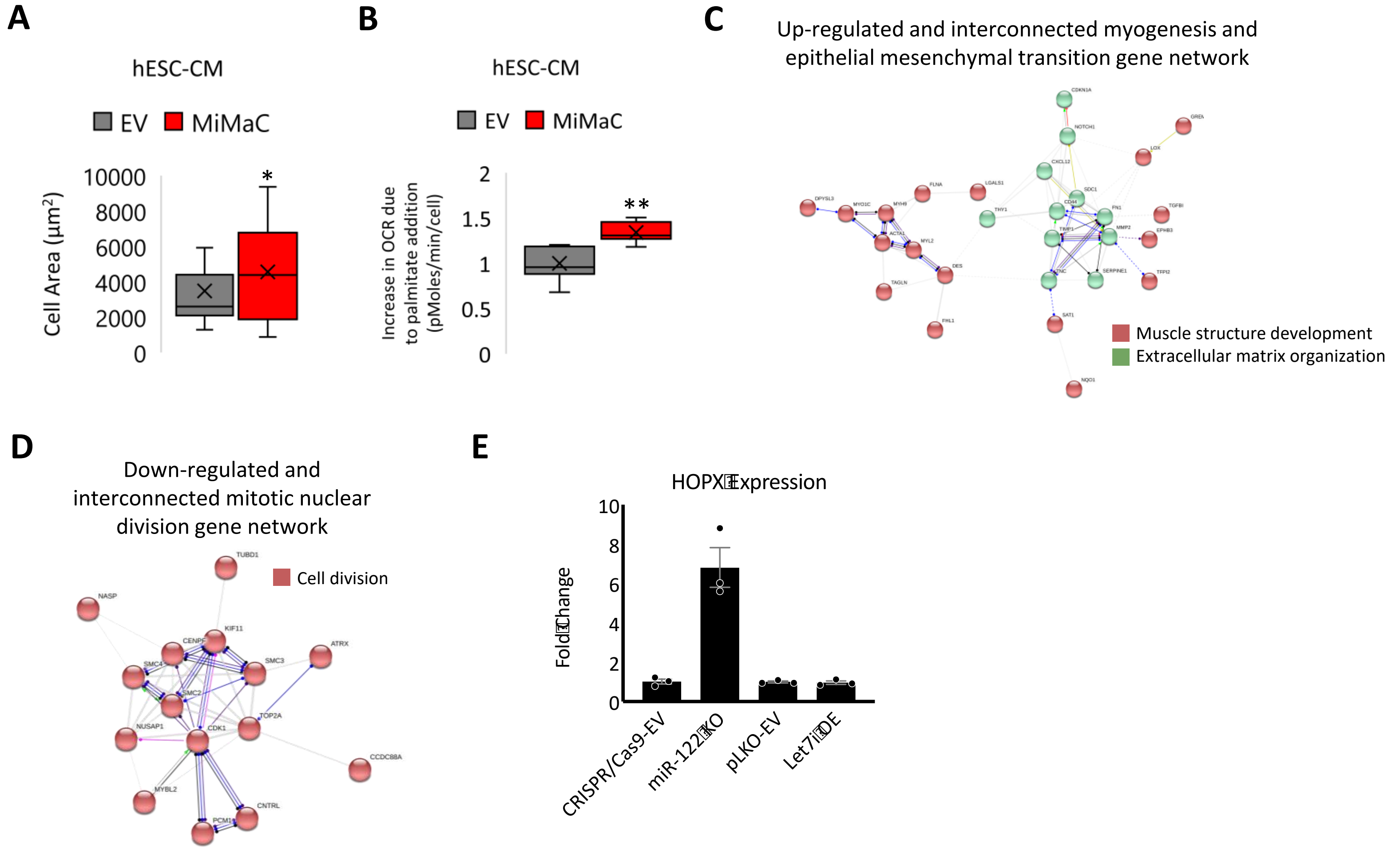

Supplemental Figure 3: MiMaC treated hPSC-CMs show an increase in cell size, ability to use fatty acids and a more mature transcriptome. A) MiMaC treated hESC-CMs showed a statistically significant increase in cell area as compared to control EV hESC-CMs. *p=0.05, t-test followed by a Mann-Whitney rank sum test. n=39-42 cells measured. B) MiMaC treated hESC-CMs were able to utilize the fatty acid palmitate to generate ATP. MiMaC treated hESC-CMs had a statistically significant increase in oxygen consumption due to the presentation of palmitate. **p<0.01, t-test was performed. n=6-7 biological replicates. C) String analysis plot of up-regulated and interconnected myogenesis and epithelial mesenchymal transition genes. Two clusters of genes are highlighted: red – muscle structure development, green – extracellular matrix organization. D) String analysis plot of down-regulated and interconnected mitotic nuclear division gene network. One cluster of genes are highlighted: red – cell division. E) Knockout of miR-122 and not Let-7i leads to up-regulation of HOPX. RT-qPCR analysis of HOPX expression in miR-122 KO hiPSC-CM and Let-7i OE hiPSC-CM and their respective controls. n=1 biological sample. Box plot middle line represents the median, x represents mean, bottom line of the box represent the median of the bottom half (1st quartile) and the top line of the box represents the median of the top half (3rd quartile). The whiskers extend from the ends of the box to the non-outlier minimum and maximum value. Bar graph shows mean with S.E. Source data are provided as a Source Data file.

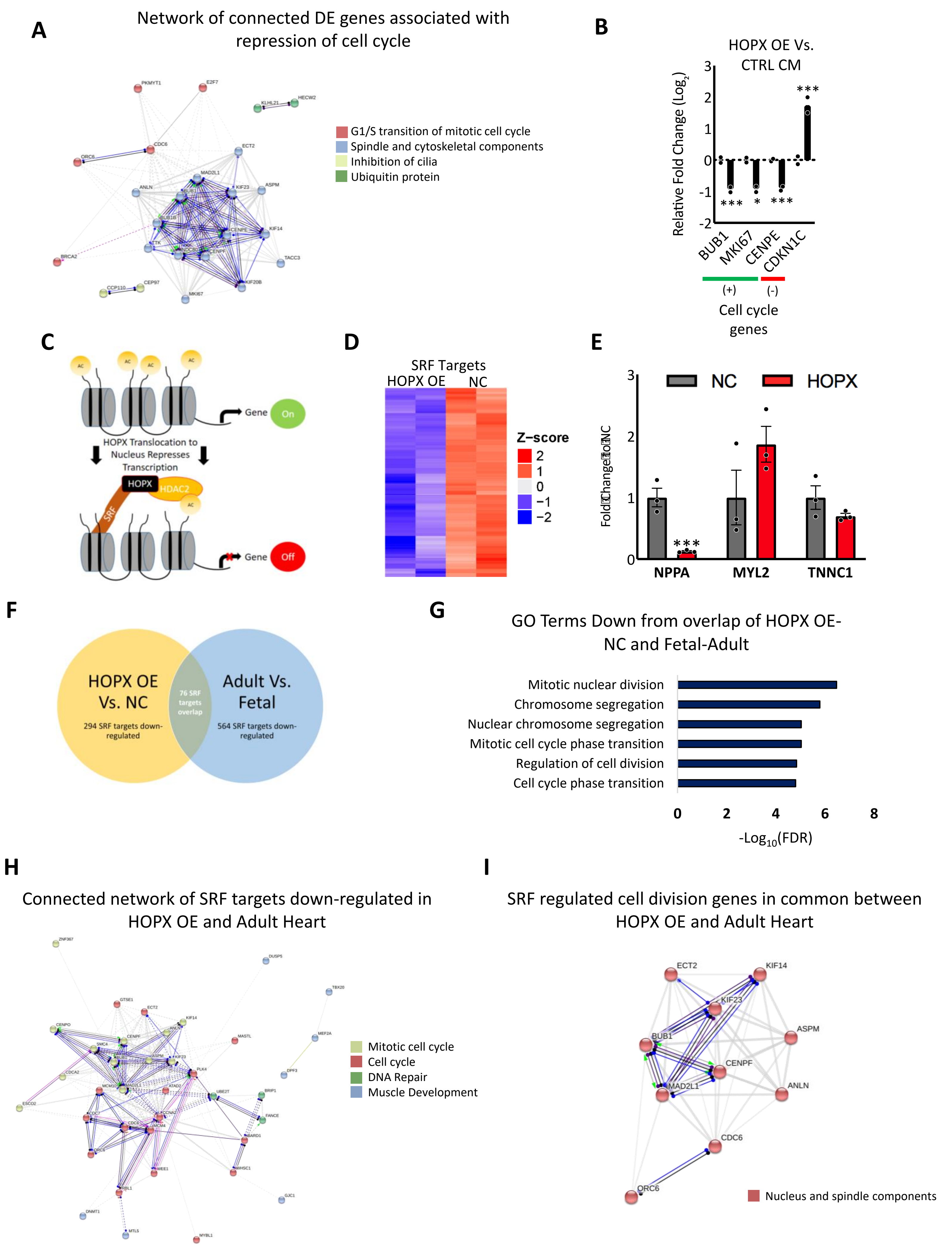
A
Network of connected DE genes associated with repression of cell cycle
G1/S transition of mitotic cell cycle
Spindle and cytoskeletal components
Inhibition of cilia
Ubiquitin protein
B
HOPX OE Vs. CTRL CM
Relative Fold Change (Log2)
BUB1
MKI67
CENPE
CDKN1C
(+)
(-)
Cell cycle genes
C
Gene
On
HOPX Translocation to Nucleus Represses Transcription
HOPX
HDAC2
SRF
Off
D
SRF Targets
HOPX OE
NC
Z-score
E
NC
HOPX
NPPA
MYL2
TNNC1
F
HOPX OE Vs. NC
294 SRF targets down-regulated
76 SRF targets overlap
Adult Vs. Fetal
564 SRF targets down-regulated
G
GO Terms Down from overlap of HOPX OE-NC and Fetal-Adult
Mitotic nuclear division
Chromosome segregation
Nuclear chromosome segregation
Mitotic cell cycle phase transition
Regulation of cell division
Cell cycle phase transition
-Log10(FDR)
H
Connected network of SRF targets down-regulated in HOPX OE and Adult Heart
Mitotic cell cycle
Cell cycle
DNA Repair
Muscle Development
I
SRF regulated cell division genes in common between HOPX OE and Adult Heart
ECT2
KIF14
KIF23
ASPM
BUB1
CENPF
MAD2L1
ANLN
CDC6
ORC6
Nucleus and spindle components

Supplemental Figure 4: HOPX OE leads to down-regulation of SRF genes regulating cell cycle. A) String analysis plot of interconnected genes associated with repression of cell cycle. Four clusters of genes are highlighted: red – G1/S transition of mitotic cell cycle, blue – spindle and cytoskeletal components, yellow – inhibition of cilia and green – ubiquitin protein. B) Fold change of cell cycle genes from RNA-Sequencing data for HOPX OE vs. NC hiPSC-CMs. Green bar represents positive regulators of cell cycle while the red bar represents negative regulators of cell cycle. ****p*<0.001, **p*<0.05, negative binomial test was performed. N=2 biological replicates. C) Schematic of how HOPX is localized to DNA via serum response factor (SRF) to then recruit histone deacetylase such as HDAC2 to repress SRF genes by removing acetylation marks. D) Heatmap showing the down-regulation of SRF targets in the HOPX OE group as compared to NC hiPSC-CMs. E) RT-qPCR analysis of NC hiPSC-CM and HOPX OE hiPSC-CM showing a significant down-regulation of gene NPPA. ****p*<0.001, t-test was performed. n=3 biological replicates. F) Venn diagram showing the number of SRF down-regulated genes in the HOPX OE vs. NC group, the human adult ventricular myocardium vs. fetal myocardium and the overlap of SRF-down-regulated genes between these two transitions. G) List of down-regulated GO terms for the overlap of HOPX OE vs. NC hiPSC-CMs and human adult vs. fetal ventricle SRF targets. H) String analysis plot of down-regulated and interconnected SRF targets in HOPX OE and human adult ventricle. Four clusters of genes are highlighted: yellow – mitotic cell cycle, red – cell cycle, green – DNA repair and blue – muscle development. I) String analysis plot of down-regulated SRF cell division genes in common between HOPX OE and human adult ventricle. One cluster was highlight: red – nucleus and spindle components. Bar graphs show mean with S.E. Source data are provided as a Source Data file.

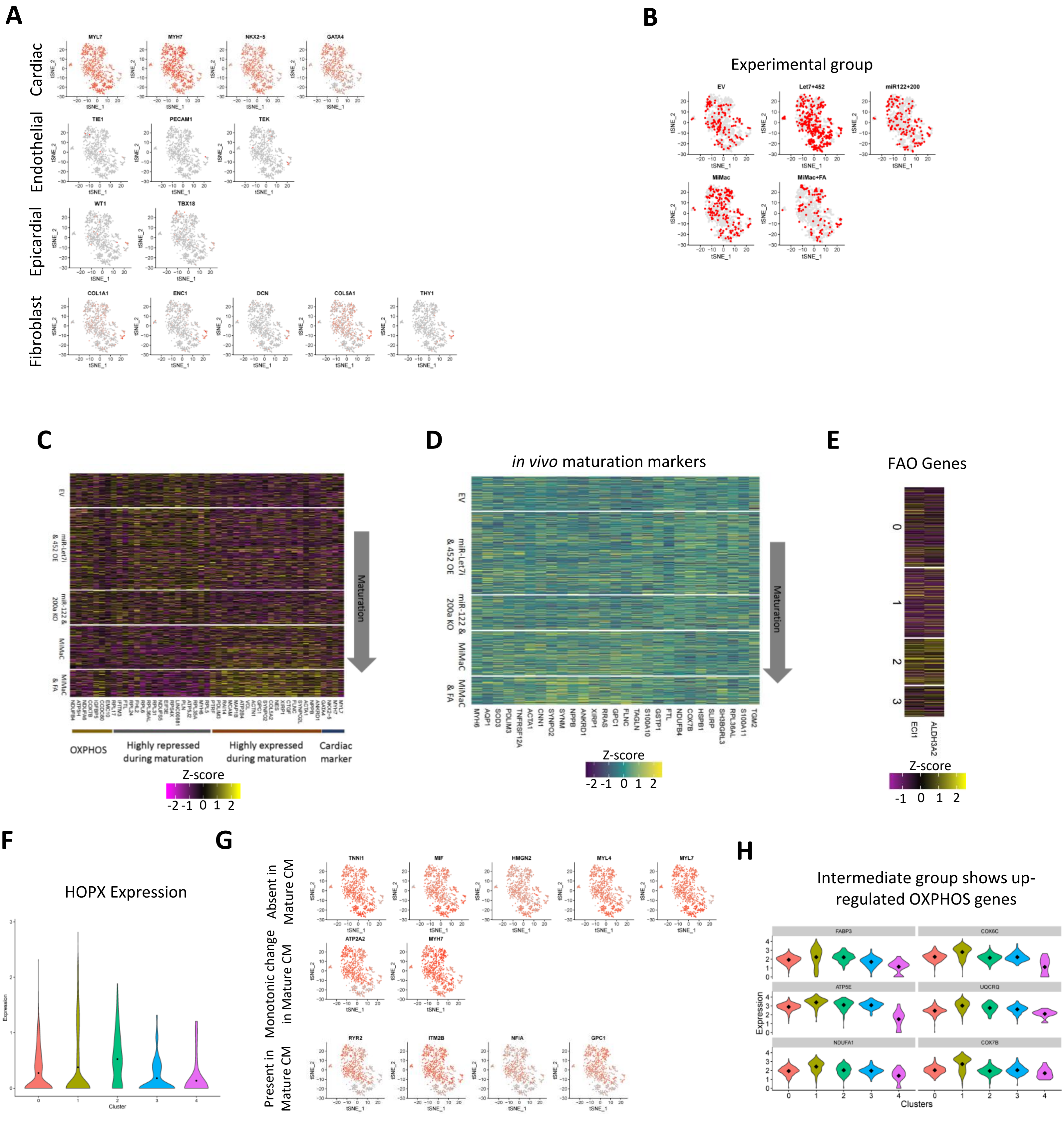

Supplemental Figure 5: MiMaC treated hPSC-CMs show a more mature transcriptome. A) tSNE plots of cell lineage markers show the majority of cells are cardiomyocytes. B) tSNE plot of each experimental group. C) Heatmap of maturation genes for each experimental group. D) Heatmap of *in vivo* human cardiac maturation markers. E) Heatmap of fatty acid oxidation genes. F) Violin plot of HOPX expression. G) Set of genes that change during cardiomyocyte maturation show the intermediate cluster is less mature. H) Set of OXPHOS genes up-regulated in cluster 2, the intermediate maturation group.

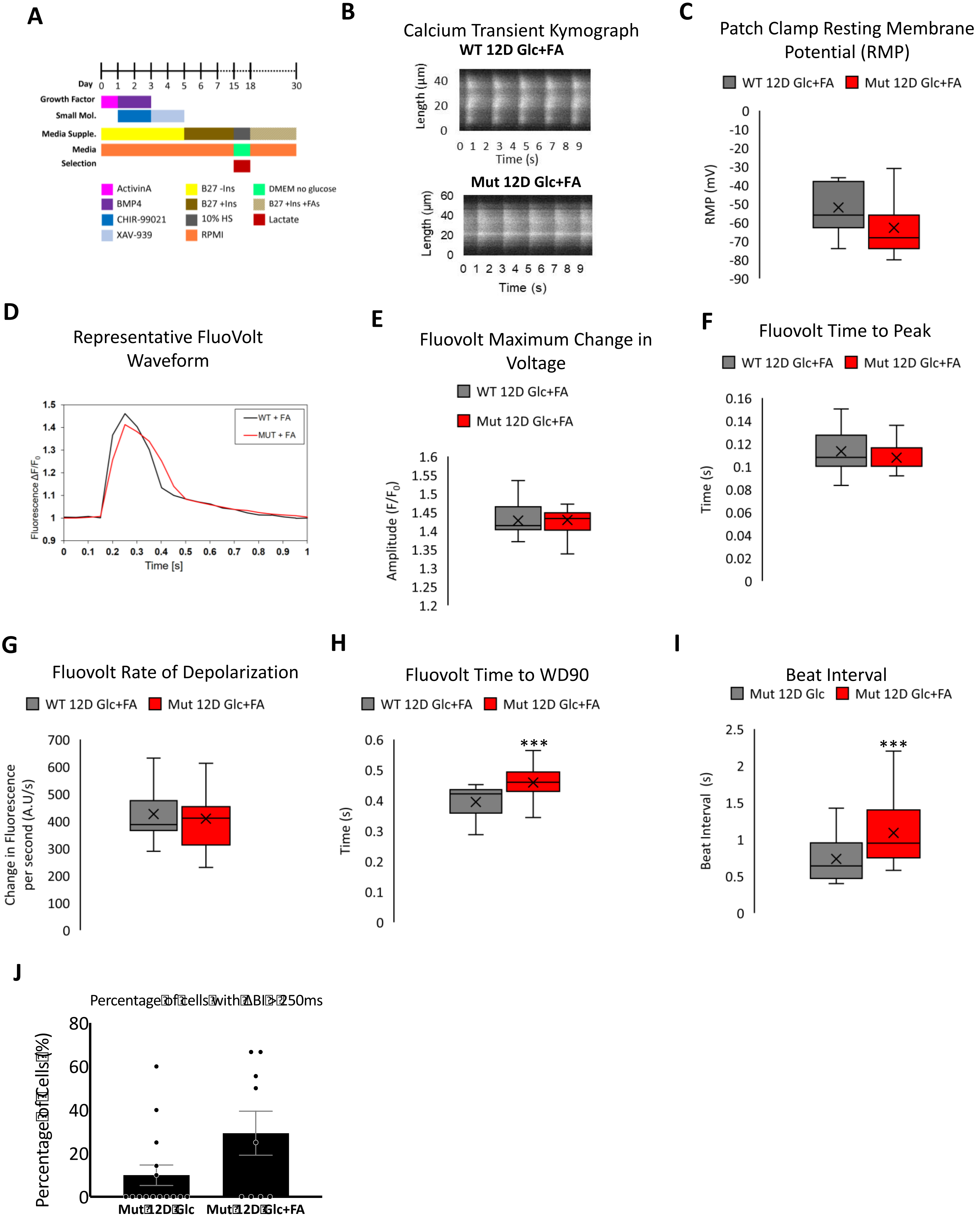
A
Day 0 1 2 3 4 5 6 7 15 18 30
Growth Factor
Small Mol.
Media Supple.
Media
Selection
ActivinA
BMP4
CHIR-99021
XAV-939
B27 -Ins
B27 +Ins
10% HS
RPMI
DMEM no glucose
B27 +Ins +FAs
Lactate
B
Calcium Transient Kymograph
WT 12D Glc+FA
Length (µm)
Time (s)
Mut 12D Glc+FA
C
Patch Clamp Resting Membrane Potential (RMP)
WT 12D Glc+FA
Mut 12D Glc+FA
RMP (mV)
D
Representative FluoVolt Waveform
WT + FA
MUT + FA
Fluorescence ΔF/F0
Time [s]
E
Fluovolt Maximum Change in Voltage
WT 12D Glc+FA
Mut 12D Glc+FA
Amplitude (F/F0)
F
Fluovolt Time to Peak
WT 12D Glc+FA
Mut 12D Glc+FA
Time (s)
G
Fluovolt Rate of Depolarization
WT 12D Glc+FA
Mut 12D Glc+FA
Change in Fluorescence per second (A.U/s)
H
Fluovolt Time to WD90
WT 12D Glc+FA
Mut 12D Glc+FA
Time (s)
***
I
Beat Interval
Mut 12D Glc
Mut 12D Glc+FA
Beat Interval (s)
***
J
Percentage of cells with ΔBI > 250ms
Percentage of Cells (%)
Mut 12D Glc
Mut 12D Glc+FA

Supplemental Figure 6: HADHA Mut hiPSC-CMs have abnormal calcium, electrophysiology and beat rates. A) Schematic of fatty acid treatment of WT and Mut hiPSC-CMs. B) Representative kymograph of WT and Mut CMs after 12D of Glc+FA media C) Quantification of the resting membrane potential from whole cell patch clamp. n=9-10 cells measured. D) Representative trace of the change in fluorescence during an action potential of WT or Mut CMs treated with Glc+FA using Fluovolt. E) Quantification of the maximum change in voltage measured by the relative maximum change in fluorescence. n=18-36 cells measured. F) Quantification of the time to reach maximum depolarization. n=9-10 cells measured. G) Quantification of the rate of maximum depolarization. n=9-10 cells measured. H) Time to wave duration 90% is significantly longer in Mut CMs as compared to WT CMs after 12D of Glc+FA media treatment. ***$p<0.001$, t-test was performed. n=9-10 cells measured. I) Quantification of the beat interval. Mut CMs in Glc+FA media as compared to Mut CMs in Glc media had a statistically significant higher beat interval. ***$p<0.001$, t-test followed by a Mann-Whitney rank sum test. n=13-16 cells measured. J) Quantification of the number of cells with a ΔBI greater than 250 ms suggesting an erratically beating cell. Mut CMs after 12D of Glc+FA media as compared to Mut CMs in Glc media had a higher percentage of cells with ΔBI's greater than 250 ms. n=9-15 cells measured. Box plot middle line represents the median, x represents mean, bottom line of the box represents the median of the bottom half (1st quartile) and the top line of the box represents the median of the top half (3rd quartile). The whiskers extend from the ends of the box to the non-outlier minimum and maximum value. Bar graph shows mean with S.E. Source data are provided as a Source Data file.

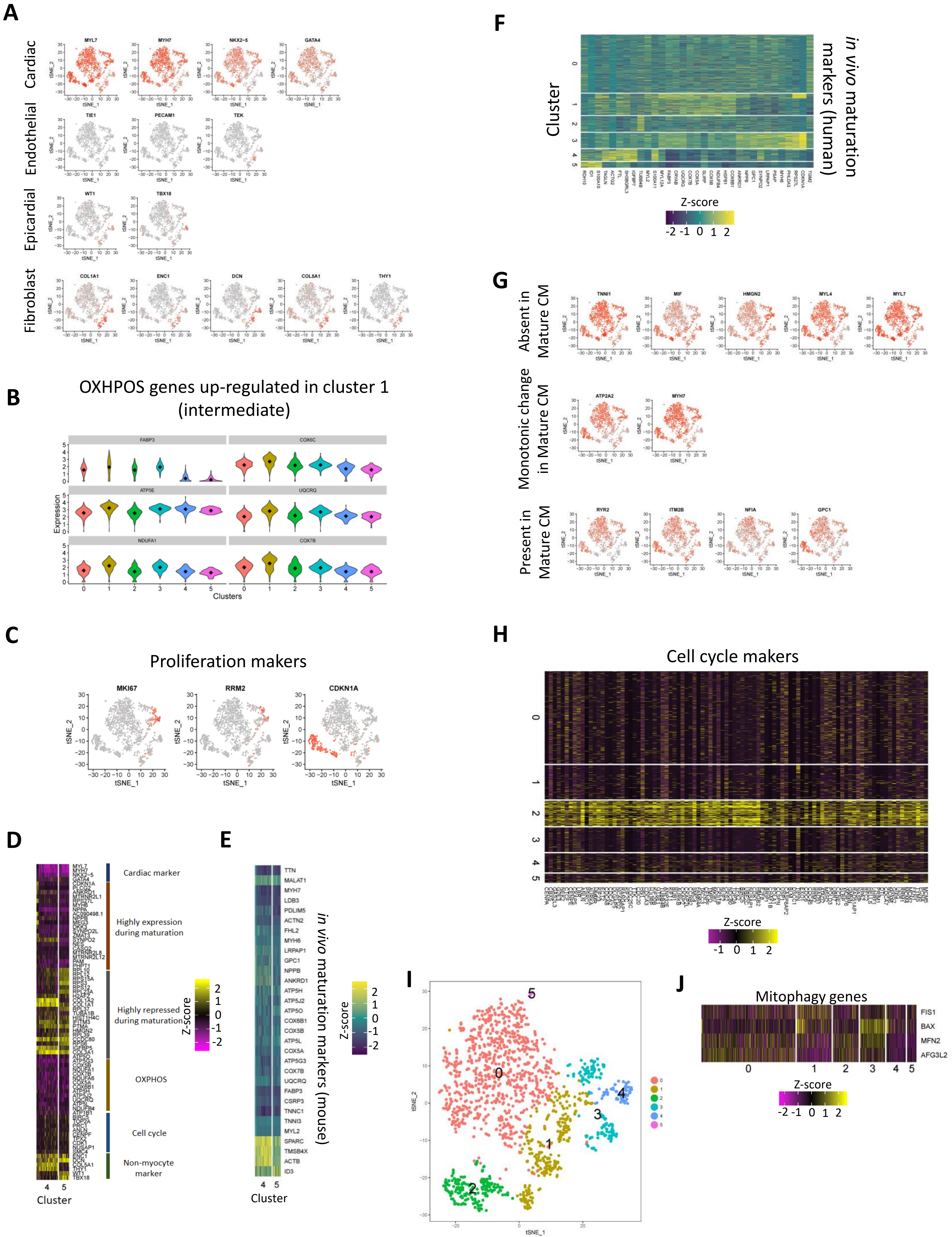

A
Cardiac
MYL7
MYH7
NKX2-5
GATA4
Endothelial
TIE1
PECAM1
TEK
Epicardial
WT1
TBX18
Fibroblast
COL1A1
ENC1
DCN
COL5A1
THY1
tSNE_1
tSNE_2
B
OXHPOS genes up-regulated in cluster 1 (intermediate)
FABP3
COX6C
ATP5E
UQCRQ
NDUFA1
COX7B
Expression
Clusters
C
Proliferation makers
MKI67
RRM2
CDKN1A
D
Cardiac marker
Highly expression during maturation
Highly repressed during maturation
OXPHOS
Cell cycle
Non-myocyte marker
Z-score
Cluster
E
TTN
MALAT1
MYH7
LDB3
PDLIM5
ACTN2
FHL2
MYH6
LRPAP1
GPC1
NPPB
ANKRD1
ATP5H
ATP5J2
ATP5O
COX6B1
COX5B
ATP5L
COX5A
ATP5G3
COX7B
UQCRQ
FABP3
CSRP3
TNNC1
TNNI3
MYL2
SPARC
TMSB4X
ACTB
ID3
in vivo maturation markers (mouse)
Z-score
Cluster
F
Cluster
in vivo maturation markers (human)
Z-score
G
Absent in Mature CM
TNNI1
MIF
HMGN2
MYL4
MYL7
Monotonic change in Mature CM
ATP2A2
MYH7
Present in Mature CM
RYR2
ITM2B
NFIA
GPC1
H
Cell cycle makers
Z-score
I
J
Mitophagy genes
FIS1
BAX
MFN2
AFG3L2
Z-score

Supplemental Figure 7: Single Cell RNA-Sequencing elucidates HADHA CM subpopulations. A) tSNE plots of cell lineage markers. B) Set of OXPHOS genes up-regulated in cluster 2, the intermediate maturation group. C) tSNE plots showing HADHA Mut CMs lose cell cycle repressor CDKN1A and a subset of HADHA Mut CMs gain markers for proliferation: MKI67 and RRM2. D) Heatmap of maturation categories based on MiMaC cluster. E) Heatmap of *in vivo* mouse maturation markers that are up-regulated during mouse cardiac maturation. F) Heatmap of *in vivo* maturation markers that are up-regulated during human cardiac maturation. G) Set of genes changing during cardiomyocyte maturation show the intermediate cluster is less mature. H) Heatmap of cell cycle genes. I) Unbiased clustering of WT and HADHA Mut CMs after the removal of cell cycle genes. J) Heatmap of mitophagy genes.

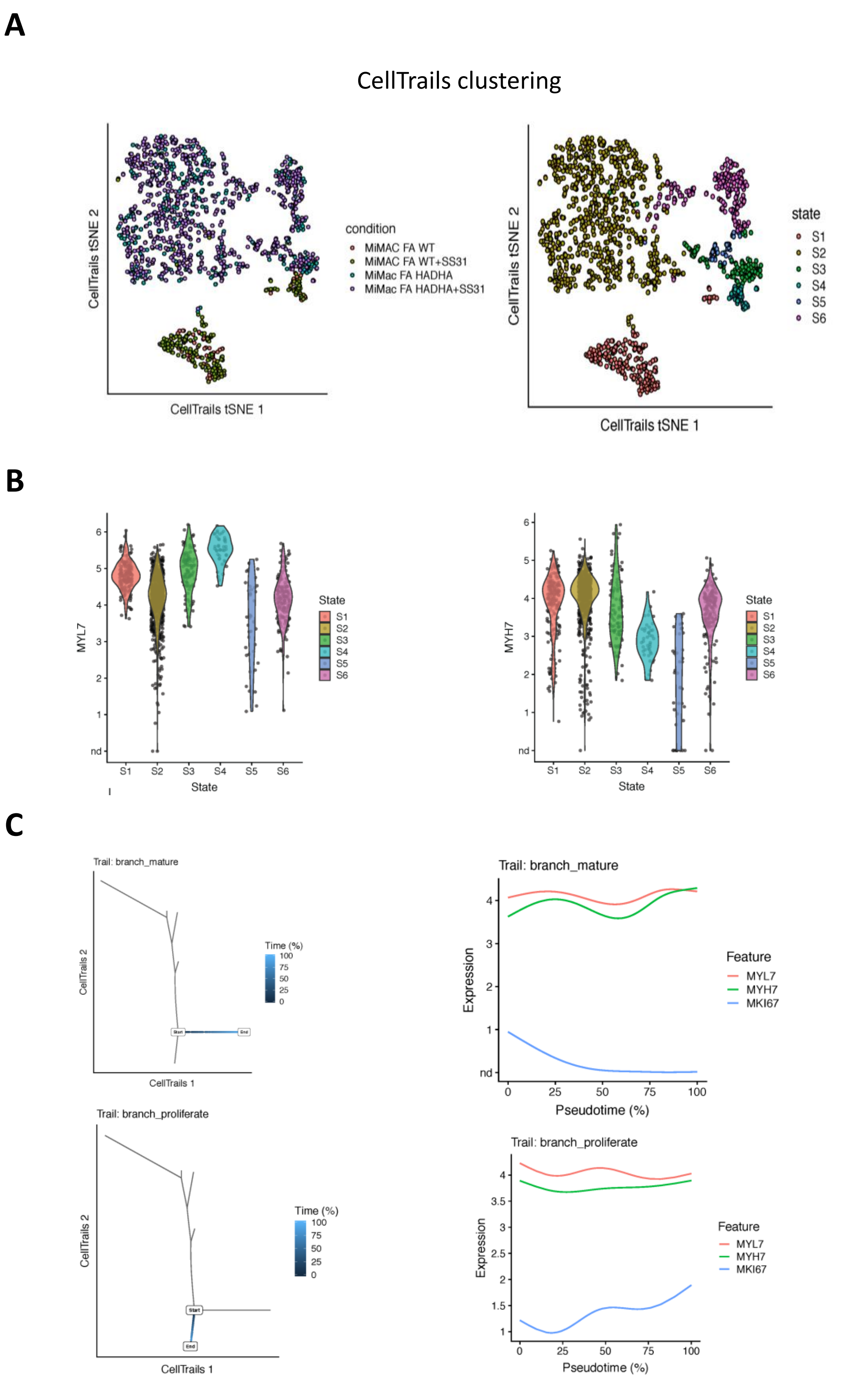

Supplemental Figure 8: WT-CMs follow a path to disease state via an intermediate state. A) tSNE plots showing CellTrails clustering based on group (left) and state (right). B) Violin plot of the maturation genes that go down with maturation (*MYL7*, left) and up with maturation (*MYH7*, right). C) CellTrails branching pathway showing the final two branching paths. HADHA Mut CMs that are non-replicating follow a path to the right with no up-regulation of the cell proliferation gene *Ki67*. HADHA Mut CMs that are replicating/endoreplicating follow at path that continues down with an up-regulation of the cell proliferation gene *Ki67*.

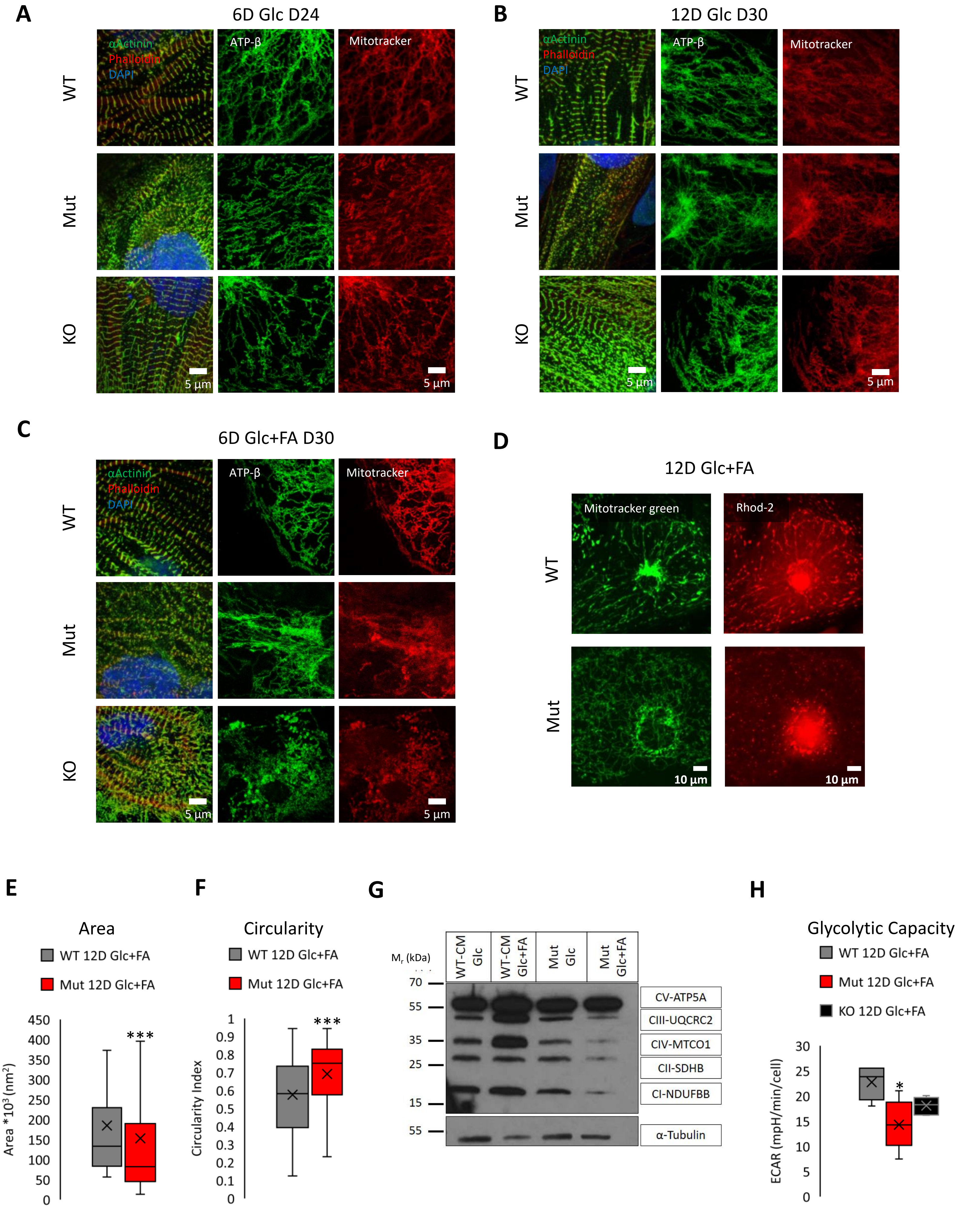
A
6D Glc D24
αActinin
Phalloidin
DAPI
ATP-β
Mitotracker
WT
Mut
KO
5 µm
5 µm
B
12D Glc D30
αActinin
Phalloidin
DAPI
ATP-β
Mitotracker
WT
Mut
KO
5 µm
5 µm
C
6D Glc+FA D30
αActinin
Phalloidin
DAPI
ATP-β
Mitotracker
WT
Mut
KO
5 µm
5 µm
D
12D Glc+FA
Mitotracker green
Rhod-2
WT
Mut
10 µm
10 µm
E
Area
WT 12D Glc+FA
Mut 12D Glc+FA
Area *10³ (nm²)
0
50
100
150
200
250
300
350
400
450
***
F
Circularity
WT 12D Glc+FA
Mut 12D Glc+FA
Circularity Index
0
0.1
0.2
0.3
0.4
0.5
0.6
0.7
0.8
0.9
1
***
G
Mr (kDa)
WT-CM Glc
WT-CM Glc+FA
Mut Glc
Mut Glc+FA
70
55
35
25
15
55
CV-ATP5A
CIII-UQCRC2
CIV-MTCO1
CII-SDHB
CI-NDUFBB
α-Tubulin
H
Glycolytic Capacity
WT 12D Glc+FA
Mut 12D Glc+FA
KO 12D Glc+FA
ECAR (mpH/min/cell)
0
5
10
15
20
25
30
*

Supplemental Figure 9: HADHA Mut hiPSC-CMs after 6D of glucose and fatty acid media treatment show mild sarcomere and mitochondrial defects. A) Representative confocal images of D24 WT, HADHA Mut and HADHA KO hiPSC-CMs cultured in glucose media. Myofibril staining of αActinin (Green) and actin (phalloidin – red) show no abnormalities. Mitochondrial staining with ATP synthase β subunit (green) and mitochondrial potential gradient shown via mitotracker staining (red) show no mitochondrial abnormalities. B) Representative confocal images of D30 WT, HADHA Mut and HADHA KO hiPSC-CMs cultured in glucose media. Myofibril staining of αActinin (Green) and actin (phalloidin – red) show no abnormalities. Mitochondrial staining with ATP synthase β subunit (green) and mitochondrial potential gradient shown via mitotracker staining (red) show no mitochondrial abnormalities. C) Representative confocal images of WT, HADHA Mut and KO hiPSC-CMs after 6D of glucose and fatty acid media treatment. After 6D of glucose and fatty acid media treatment WT hiPSC-CMs display normal sarcomere and mitochondria. After 6D of glucose and fatty acid media treatment HADHA Mut and KO hiPSC-CMs display signs of sarcomere disruption as seen in the less defined αActinin staining and the beginnings of loss of mitochondrial proton gradient as seen from the mitotracker staining in the mutant. ATP synthase β subunit (green) shows normal mitochondrial networks in both the WT and HADHA Mut hiPSC-CMs while there are the beginnings of loss of mitochondrial network in the HADHA KO hiPSC-CMs. D) Representative images of mitotracker green and Rhod2 dye in WT and Mut CMs after 12D of Glc+FA treatment. E) Quantification of mitochondria area in WT and HADHA Mut CMs shows small mitochondria size in Mut CMs. ***$p<0.001$, t-test followed by a Mann-Whitney rank sum test. n=81-236 mitochondria measured. F) Quantification of mitochondria circularity index in WT and HADHA Mut CMs shows rounder mitochondria in Mut CMs. ***$p<0.001$, t-test followed by a Mann-Whitney rank sum test. n=81-236 mitochondria measured. G) HADHA Mut hiPSC-CMs show a reduction in complex I-IV proteins after 12D of glucose and fatty acid media treatment. Western analysis of complex I-V of the electron transport chain for WT and HADHA Mut hiPSC-CMs cultured either in glucose or glucose and fatty acids for 12D. H) Quantification of glycolytic capacity from mitostress assay, calculated as the difference between the extracellular acidification rate after oligomycin and 2-deoxy-D-glucose. Mut CMs as compared to WT CMs after 12D of Glc+FA media had significantly lower glycolytic capacity. *$p<0.05$, one-way ANOVA on ranks was performed vs WT 12D Glc+FA. n=6-19 biological replicates. Box plot middle line represents the median, x represents mean, bottom line of the box represents the median of the bottom half (1st quartile) and the top line of the box represents the median of the top half (3rd quartile). The whiskers extend from the ends of the box to the non-outlier minimum and maximum value. Source data are provided as a Source Data file.

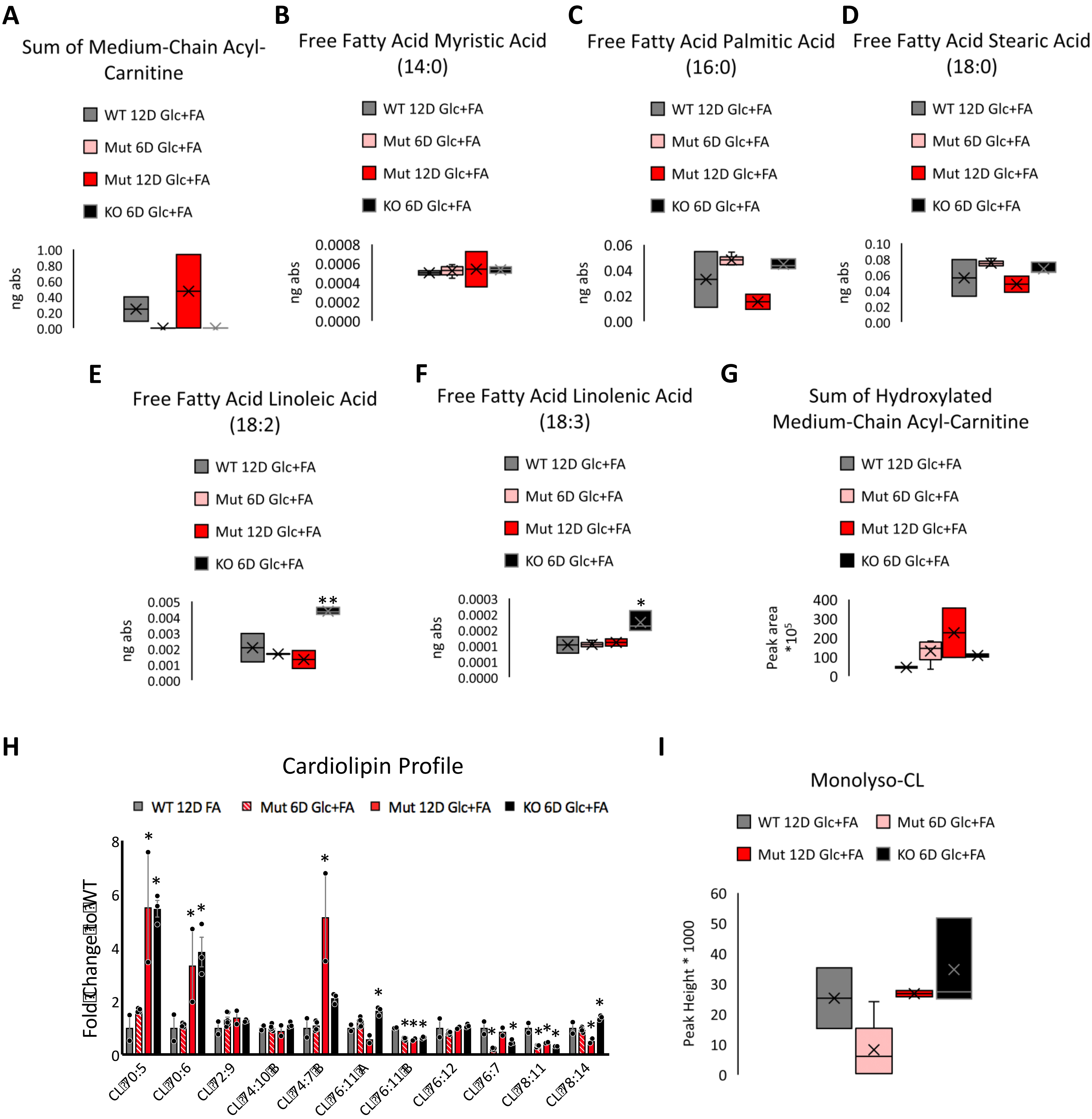

A
Sum of Medium-Chain Acyl-Carnitine
WT 12D Glc+FA
Mut 6D Glc+FA
Mut 12D Glc+FA
KO 6D Glc+FA
ng abs
B
Free Fatty Acid Myristic Acid (14:0)
C
Free Fatty Acid Palmitic Acid (16:0)
D
Free Fatty Acid Stearic Acid (18:0)
E
Free Fatty Acid Linoleic Acid (18:2)
F
Free Fatty Acid Linolenic Acid (18:3)
G
Sum of Hydroxylated Medium-Chain Acyl-Carnitine
Peak area *10^5
H
Cardiolipin Profile
WT 12D FA
Mut 6D Glc+FA
Mut 12D Glc+FA
KO 6D Glc+FA
Fold Change to WT
CL 70:5
CL 70:6
CL 72:9
CL 74:10B
CL 74:7B
CL 76:11A
CL 76:11B
CL 76:12
CL 76:7
CL 78:11
CL 78:14
I
Monolyso-CL
Peak Height * 1000

Supplemental Figure 10: HADHA Mut and KO CMs have abnormal CL remodeling. A) The sum of all medium-chain acyl-carnitines in WT, HADHA Mut and HADHA KO FA treated hPSC-CMs. n=2-6 biological replicates. B) Amount of myristic acid (14:0) in the free fatty acid state in WT, HADHA Mut and HADHA KO FA treated hPSC-CMs. n=2-6 biological replicates. C) Amount of palmitic acid (16:0) in the free fatty acid state in WT, HADHA Mut and HADHA KO FA treated hPSC-CMs. n=2-6 biological replicates. D) Amount of stearic acid (18:0) in the free fatty acid state in WT, HADHA Mut and HADHA KO FA treated hPSC-CMs. n=2-6 biological replicates. E) Amount of linoleic acid (18:2) in the free fatty acid state in WT, HADHA Mut and HADHA KO FA treated hPSC-CMs. ** $p<0.01$, one-way ANOVA was performed vs WT 12D Glc+FA. n=2-6 biological replicates. F) Amount of linolenic acid (18:3) in the free fatty acid state in WT, HADHA Mut and HADHA KO FA treated hPSC-CMs. * $p<0.05$, one-way ANOVA was performed vs WT 12D Glc+FA. n=2-6 biological replicates. G) Sum of all identified hydroxylated medium-chain acyl-carnitine species. n=2-6 biological replicates. H) Fold change differences to WT FA CM for cardiolipin species in WT and HADHA Mut CMs from global lipidomics. # $p=0.051$, * $p<0.05$, ** $p<0.01$, *** $p<0.001$, one-way ANOVA was performed vs WT 12D Glc+FA. n=2-6 biological replicates. I) The relative abundance of MLCL in WT, HADHA Mut and HADHA KO FA treated hPSC-CMs. n=2-6 biological replicates. Box plot middle line represents the median, x represents mean, bottom line of the box represents the median of the bottom half (1st quartile) and the top line of the box represents the median of the top half (3rd quartile). The whiskers extend from the ends of the box to the non-outlier minimum and maximum value. Bar graph shows mean with S.E. Source data are provided as a Source Data file.
