# Supplementary material for: TFPa/HADHA is required for fatty acid beta-oxidation and cardiolipin re-modeling in human cardiomyocytes
Source: Nat Commun. 2019 Oct 11;10:4671. doi: 10.1038/s41467-019-12482-1 (PMC6789043; doi:10.1038/s41467-019-12482-1)
Supplement: Supplementary file 3 — Reporting Summary [file 41467_2019_12482_MOESM3_ESM.pdf]

nature research

Corresponding author(s): Hannele Ruohola-Baker

Reporting Summary

Nature Research wishes to improve the reproducibility of the work that we publish. This form provides structure for consistency and transparency in reporting. For further information on Nature Research policies, see Authors & Referees and the Editorial Policy Checklist.

Statistical parameters

When statistical analyses are reported, confirm that the following items are present in the relevant location (e.g. figure legend, table legend, main text, or Methods section).

n/a	Confirmed	
☐	☒	The exact sample size (*n*) for each experimental group/condition, given as a discrete number and unit of measurement
☐	☒	An indication of whether measurements were taken from distinct samples or whether the same sample was measured repeatedly
☐	☒	The statistical test(s) used AND whether they are one- or two-sided<br>*Only common tests should be described solely by name; describe more complex techniques in the Methods section.*
☒	☐	A description of all covariates tested
☐	☒	A description of any assumptions or corrections, such as tests of normality and adjustment for multiple comparisons
☐	☒	A full description of the statistics including central tendency (e.g. means) or other basic estimates (e.g. regression coefficient) AND variation (e.g. standard deviation) or associated estimates of uncertainty (e.g. confidence intervals)
☐	☒	For null hypothesis testing, the test statistic (e.g. *F*, *t*, *r*) with confidence intervals, effect sizes, degrees of freedom and *P* value noted<br>*Give P values as exact values whenever suitable.*
☒	☐	For Bayesian analysis, information on the choice of priors and Markov chain Monte Carlo settings
☒	☐	For hierarchical and complex designs, identification of the appropriate level for tests and full reporting of outcomes
☒	☐	Estimates of effect sizes (e.g. Cohen's *d*, Pearson's *r*), indicating how they were calculated
☐	☒	Clearly defined error bars<br>*State explicitly what error bars represent (e.g. SD, SE, CI)*

Our web collection on statistics for biologists may be useful.

Software and code

Policy information about availability of computer code

Data collection	*Provide a description of all commercial, open source and custom code used to collect the data in this study, specifying the version used OR state that no software was used.*
Data analysis	Matlab<br>Tophat (RNA-Seq)<br>R (Princomp, TopGO package, Seurat)<br>CellRanger (scRNA-Seq)<br>STRING Analysis (online software for protein interaction maps)<br>MassHunter Workstation Software Quantitative Analysis for QQQ B.07.00 (Agilent)<br>MS-DIAL (Untargeted lipidomic)<br>CellTrails

For manuscripts utilizing custom algorithms or software that are central to the research but not yet described in published literature, software must be made available to editors/reviewers upon request. We strongly encourage code deposition in a community repository (e.g. GitHub). See the Nature Research guidelines for submitting code & software for further information.

Data

Policy information about availability of data

All manuscripts must include a data availability statement. This statement should provide the following information, where applicable:

- Accession codes, unique identifiers, or web links for publicly available datasets
- A list of figures that have associated raw data
- A description of any restrictions on data availability

GSE135595, NIH Metabolomics Workbench Study ID: ST001246

Field-specific reporting

Please select the best fit for your research. If you are not sure, read the appropriate sections before making your selection.

☒ Life sciences ☐ Behavioural & social sciences ☐ Ecological, evolutionary & environmental sciences

For a reference copy of the document with all sections, see nature.com/authors/policies/ReportingSummary-flat.pdf

Life sciences study design

All studies must disclose on these points even when the disclosure is negative.

Sample size	We chose a sample size of 3 for the majority of the experiments.
Data exclusions	No data exclusions.
Replication	Findings were repeated in biological replicates. When we did this, we found similar results between the different experiments.
Randomization	This was not relevant to our study.
Blinding	The investigators were not blinded. Many of the assays performed in this study were non-biased, as we used computation methods to analyze data sets or we had collaborators assess functional aspects of the cells, such as force of contraction, calcium handling etc.

Reporting for specific materials, systems and methods

Materials & experimental systems

n/a	Involved in the study
☒	☐ Unique biological materials
☐	☒ Antibodies
☐	☒ Eukaryotic cell lines
☒	☐ Palaeontology
☒	☐ Animals and other organisms
☐	☒ Human research participants

Methods

n/a	Involved in the study
☒	☐ ChIP-seq
☒	☐ Flow cytometry
☒	☐ MRI-based neuroimaging

Antibodies

Antibodies used

Antibodies for Westerns: Alpha tubulin antibody Cell Signalling Technologies (2144) 1:2000, Beta tubulin Promega (G7121) anti-mouse 1:4000, Beta Actin Cell Signalling Technologies (4970) 1:4000, HADHA Abcam (ab54477 anti-rabbit 1:1000, UCP3 Abcam (ab3477) anti-rabbit 1:200, SLC25A4 (ANT1) Sigma (SAB2105530) anti-rabbit 1:1000, OXPHOS MitoSciences (MS604/G2830) anti-mouse 1:1000, anti-GFP Invitrogen (A-11122) anti-rabbit 1:1000.

Antibodies for IHC: αActinin 1:250 Sigma A7811 anti-mouse, HADHA 1:250 abcam ab54477 anti-rabbit, ATP Synthase β 1:250 abcam ab14730 anti-mouse, Titin 1:300 Myomedix TTN-9 (cTerm) anti-rabbit, GFP 1:300 Invitrogen A-11122 anti-rabbit. Secondary antibodies and other reagents used were: DAPI at a concentration of 0.02µg/mL, phalloidin alexa fluor 568 1:250, alexa fluor 488 or 647-conjugated goat anti-mouse and anti-rabbit secondary antibodies 1:500 (Molecular Probes).

Validation

These are common antibodies, used following the manufactures recommended parameters.

Eukaryotic cell lines

Policy information about cell lines

Cell line source(s)

RUES2 (NIHhESC-09-0013) (WiCell, RUESe002-A)
hiPSC line WTC #11, previously derived in the Conklin laboratory at UCSF (Coriell Institute, GM25256)
hiPSC line HEL87.1, previously derived in the Suomalainen laboratory at University of Helsinki

Authentication

For HEL87.1 we confirmed the point mutation at c.1528G<C. For the RUES2 and WTC #11 lines, we did not further authenticate the lines more than the original creators of the lines.

Mycoplasma contamination

Mycoplasma contamination was not performed

Commonly misidentified lines
(See ICLAC register)

Name any commonly misidentified cell lines used in the study and provide a rationale for their use.

Human research participants

Policy information about studies involving human research participants

Population characteristics

Describe the covariate-relevant population characteristics of the human research participants (e.g. age, gender, genotypic information, past and current diagnosis and treatment categories). If you filled out the behavioural & social sciences study design questions and have nothing to add here, write "See above."

Recruitment

The patient manifested the LCHAD deficiency disease during the first months after birth with hypoketotic hypoglycemia and failure to thrive, with metabolic acidosis, cardiomyopathy and hepatomegaly. The skin sampling to obtain fibroblast cultures was performed with informed consent of the parents, as approved by the ethical review board of Helsinki University Hospital, and according to Helsinki Declaration.
